# Possible future trajectory of COVID-19: emphasis on Africa

**DOI:** 10.11604/pamj.2021.40.157.31905

**Published:** 2021-11-16

**Authors:** Seth Kwabena Amponsah, Benjamin Tagoe, Daniel Kwame Afriyie

**Affiliations:** 1 Department of Medical Pharmacology, University of Ghana Medical School, Accra, Ghana,; 2 Fulfilment Operations and Academy, Zipline Ghana, Accra, Ghana,; 3 Ghana Police Hospital, Accra, Ghana

**Keywords:** COVID-19, Africa, vaccine, trajectory

## Abstract

Coronavirus disease 2019 (COVID-19) has caused morbidity and mortality in many countries. COVID-19 has also negatively affected the economy of several nations. The dynamics of interaction between severe acute respiratory syndrome coronavirus 2 (SARS-CoV-2) and host, and possible evolution of the virus into more virulent strains pose a threat to global eradication. With the advent of vaccination in most countries, vaccine hesitancy, especially in Africa, is expected to reduce. We also believe that the COVID-19 vaccine would have substantial impact on reducing incidence, hospitalizations, and deaths. A predictor model for COVID-19 infection pattern through to 2025 suggests that recurrent outbreaks are likely to occur. There is a prediction that Africa would not fully recover from the economic crises posed by the pandemic; nonetheless, we expect that economic activities on the continent will improve as countries undertake mass vaccinations and populace attain herd immunity. The growth of e-commerce has been remarkable during the pandemic and we don´t expect trend to decline anytime soon. The pandemic has led to technology and digital platform utilization and/or improvement, which invariably has the tendency to improve quality of lives in the future. These include effective big data monitoring, online shopping, among others. Our future trajectory for recurrent waves of COVID-19 is that these may occur in winter months in temperate climates. We believe that COVID-19 has strengthened Africa´s resilience to future pandemics.

## Perspective

Coronavirus disease 2019 (COVID-19) is caused by severe acute respiratory syndrome coronavirus-2 (SARS-CoV-2). COVID-19 was first reported in Wuhan, China, in December 2019. The disease has since spread to almost every country in the world [1]. As at 1^st^ October 2021, the number of confirmed COVID-19 cases worldwide stood at 234,667,541 with 4,799,844 deaths [2]. The African region has recorded relatively lower numbers of severe COVID-19 cases and case fatality rates. Additionally, the proportion of patients requiring intensive care management in Africa has been low [3]. COVID-19 has negatively affected the economy of many countries. United Nations Educational, Scientific and Cultural Organization (UNESCO) estimated that over 900 million learners had their academic calendars distorted and were forced to learn from home, at a point during the pandemic [4].

In some developed countries, the Centers for Disease Control and Prevention (CDC) partnered with some biomedical industries to manufacture rapid diagnostic test kits to meet high demand. Other strategies that have been employed in tackling the pandemic include lockdowns, identifying and quarantining infected individuals at early stages, community-wide education on risk factors, amongst others [5]. Currently, a number of vaccines have been developed for COVID-19. With the advent of vaccination in most countries, vaccine hesitancy, especially in Africa, is expected to reduce. Among the vaccines include Oxford/AstraZeneca, Pfizer-BioNTech and Moderna. Although these vaccines have been developed, distribution, supply chain issues and storage could be a major challenge to access especially in low- and middle-income countries. There are reports that it is unclear whether the COVID-19 vaccines developed by Moderna and Pfizer-BioNTech protect people from being infected, or from transmitting the virus to other people [6]. Thus, it is relevant to consider the new equilibrium between humans and SARS-CoV-2. The goal of this perspective is to discuss possible future trajectory of COVID-19.

**SARS-CoV-2:** SARS-CoV-2 is outpacing emergency public health responses in most countries [7]. The Delta variant of SARS-CoV-2 is a typical example of a succeeding strain, with an increased virulence. Scientific reports have predicted further evolution of the virus. The pattern of infection of SARS-CoV-2 over the past 18 months in most countries attests to this fact. There is enough reason to believe that the Delta variant mutates very rapidly. Mutation of the Delta variant could help the strain evade vaccines, and also promote multi-drug resistance to approved antiviral drugs that are to be used for prophylaxis and treatment of COVID-19. There is also the likelihood that the Delta strain could evolve into a less harmful variant that causes respiratory infections similar to flu.

A predictor model for COVID-9 infection pattern through 2025, suggests that recurrent outbreaks are likely to occur in the coming years in a similar manner to other related coronaviruses especially during winter [1]. The incidence rate of SARS-CoV-2 is likely to be significantly dependent on immunity as well as cross-immunity towards similar viruses [8]. To better predict the effectiveness of vaccines as well as post pandemic dynamics of the virus, serological testing is required to fully understand the extent and duration of immunity to SARS-CoV-2 [8,9]. We believe surveillance and intermittent social distancing should still be sustained through to 2025, to effectively control the pandemic.

**Economy:** reports suggest that a number of African countries are increasingly adopting the use of digital technology to boost productivity in jobs, and increase employment opportunities in the midst of the pandemic. Economic recovery is expected to vary significantly across countries. There is a prediction that Africa would not fully recover from the economic crises posed by the pandemic [10]. Reports also suggest that between 2021 to 2022, a 0.8% increase in economic growth is expected in Eastern and Southern Africa, whereas a 9% increase is expected in Western and Central Africa [11]. The World Bank predicts that COVID-19 could push up to 40 million people into extreme poverty, erasing at least 5 years of progress made to mitigate poverty [10]. Nonetheless, we expect that economic activities on the continent will improve as countries undertake mass vaccinations in an attempt to contain the spread of SARS-CoV-2.

**Figure 1:**
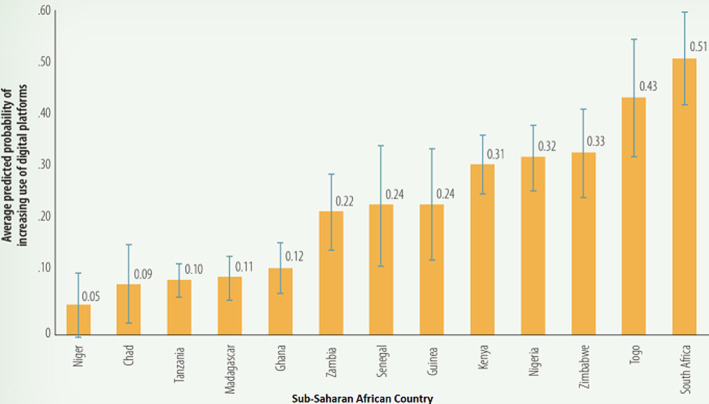
average adjusted probability of starting or increasing the use of digital technology in sub-Saharan Africa

**Digital technology:** in a survey that involved 18 African countries, the use of digital platforms and solutions in response to COVID-19 pandemic was highest in South Africa (51%) and lowest in Niger (5%) [10]. The authors predict that utilization of digital technology will increase across the continent regardless of infection pattern of COVID-19, as seen in Figure 1. Utilization of digital platforms in sub-Saharan African firms was found to be highest among financial institutions (40%) and information and communications technology (ICT) services (39%) [10]. The growth of e-commerce has been remarkable during the pandemic and we don´t expect trend to decline anytime soon.

**Vaccination:** given the limited population-level immunity to COVID-19, vaccination remains a key preventive measure to reduce disease burden and mitigate future outbreaks. There is evidence that mass vaccination will help reduce the incidence of COVID-19-induced deaths. In England, for instance, of all COVID-19-related deaths between January and July 2021, 35% of these were people who had not received COVID-19 vaccines [12]. Reports also suggest that individuals who had been vaccinated up to twice had 0.8% COVID-19-related deaths [13,14]. We also believe that the COVID-19 vaccination would have a substantial impact on reducing incidence, hospitalizations, and deaths, especially among vulnerable individuals with comorbidities and risk factors associated with severe COVID-19.

**Other possible relevant future trajectories:** in the Western world, perhaps rates of COVID-19 infection and mortality may still be high by the end of 2022. These deaths are likely to be clustered during winter months in temperate climates. However, fewer COVID-19 related deaths are expected in Africa. Furthermore, more potent vaccines are likely to be developed with mild risks of adverse reactions, and more easily incorporated into routine childhood immunization schedules [15]. There is the likelihood of a paradigm shift to focusing on other infectious and noninfectious diseases (like cancer, hypertension, asthma, heart failure and stroke). Also, lessons learnt from the COVID-19 pandemic will inform the use of big data in monitoring future pandemics [16].

We predict that pharmaceutical and scientific industries will channel most of their resources to the development of vaccines and antiviral drugs that can eradicate COVID-19 infections completely, to the neglect of other infectious and chronic diseases. In conclusion, we believe that COVID-19 has strengthened Africa´s resilience to future pandemics. Nonetheless, intermittent social distancing, serological testing and surveillance may be necessary. Additional interventions, including adequate vaccine coverage and rollout of effective therapeutic agents would improve and hasten acquisition of herd immunity.
